# Correction: Martel-Rodríguez et al. Long-Term Performance of a Hybrid-Flow Constructed Wetlands System for Urban Wastewater Treatment in Caldera de Tirajana (Santa Lucía, Gran Canaria, Spain). *Int. J. Environ. Res. Public Health* 2022, *19*, 14871

**DOI:** 10.3390/ijerph21081058

**Published:** 2024-08-13

**Authors:** Gilberto M. Martel-Rodríguez, Vanessa Millán-Gabet, Carlos A. Mendieta-Pino, Eva García-Romero, José R. Sánchez-Ramírez

**Affiliations:** 1 Water Department, Instituto Tecnológico de Canarias (ITC), 35119 Santa Lucía, Spain; vmillan@itccanarias.org; 2 Department of Process Engineering, University of Las Palmas de Gran Canaria (ULPGC), 35214 Las Palmas de Gran Canaria, Spain; carlos.mendieta@ulpgc.es; 3 Mancomunidad Intermunicipal del Sureste de Gran Canaria, 35118 Agüimes, Spain; evagarcia@surestegc.org (E.G.-R.); mancomunidad@surestegc.org (J.R.S.-R.)

## Text Correction

There was an error in the original publication [1]. A correction has been made to “3.3.3 Secondary Treatment Effluent”; “Vertical-flow constructed wetlands, VFCWs”; “(c) Effect of temperature on the effluent of the VFCWs”; Paragraph 4:

With respect to the decrease in the TSS as the temperature increases, this could be attributable to the large contribution of organic matter in its composition, the degradation of which is favored by higher temperatures. In addition, higher temperatures favor the solubility of particular substances that are present in the water, which contributes to lowering the TSS values. Finally, higher temperatures also favor the growth of Las temperaturas medias anuales oscilan entre 15 y 25 °C y superan los 30 °C durante los meses de verano, por lo que proporcionan las condiciones óptimas para muchos procesos biológicos y la actividad biológica de los microorganismos [21].

The correct paragraph should be:

With respect to the decrease in the TSS as the temperature increases, this could be attributable to the large contribution of organic matter in its composition, the degradation of which is favored by higher temperatures. In addition, higher temperatures favor the solubility of particular substances that are present in the water, which contributes to lowering the TSS values. Finally, higher temperatures also favor the growth of *Typha* spp. and root development in the wetlands, which may also be contributing to particle retention.

The authors state that the scientific conclusions are unaffected. This correction was approved by the Academic Editor. The original publication has also been updated.
