# Supplementary material for: Long-Term Performance of a Hybrid-Flow Constructed Wetlands System for Urban Wastewater Treatment in Caldera de Tirajana (Santa Lucía, Gran Canaria, Spain)
Source: Int J Environ Res Public Health. 2022 Nov 11;19(22):14871. doi: 10.3390/ijerph192214871 (PMC9690933; doi:10.3390/ijerph192214871)
Supplement: Supplementary file 1 [file ijerph-19-14871-s001.zip › Figure S2.pdf]

Supplementary information S2

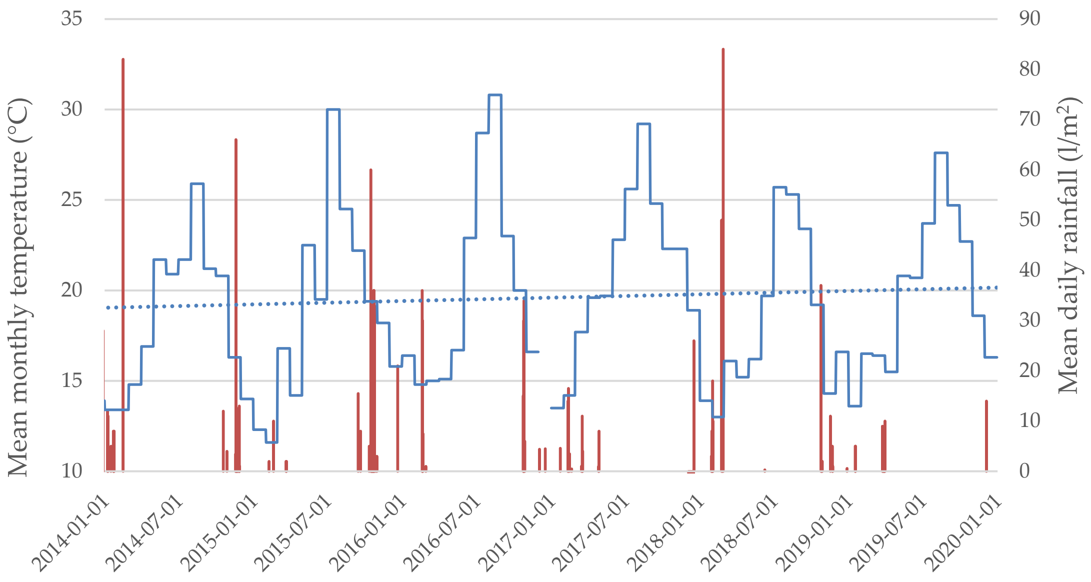

Figure S2. Mean monthly temperature (°C, blue) and mean daily rainfall (l/m^2, red) at weather station C636K
