# Supplementary material for: Long-Term Performance of a Hybrid-Flow Constructed Wetlands System for Urban Wastewater Treatment in Caldera de Tirajana (Santa Lucía, Gran Canaria, Spain)
Source: Int J Environ Res Public Health. 2022 Nov 11;19(22):14871. doi: 10.3390/ijerph192214871 (PMC9690933; doi:10.3390/ijerph192214871)
Supplement: Supplementary file 1 [file ijerph-19-14871-s001.zip › Figures S3.1 to S3.20.pdf]

Supplementary information S3

Note: for all figures, points are punctual values and dotted lines are trend lines.

Influent of the Santa Lucía HCWS

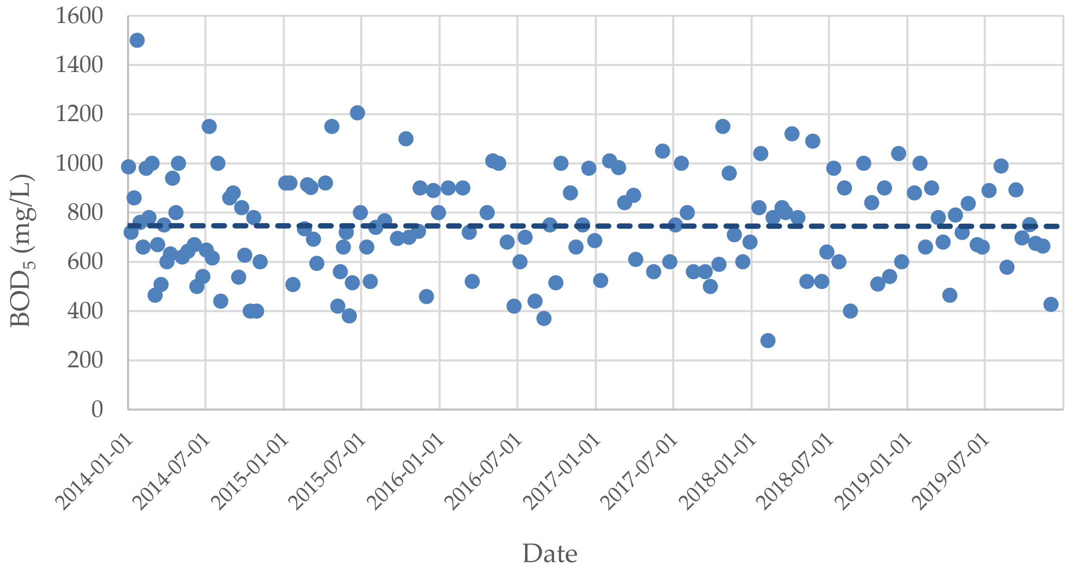

Figure S3.1. Evolution of BOD_5 in the influent of the NWWTS of Santa Lucía

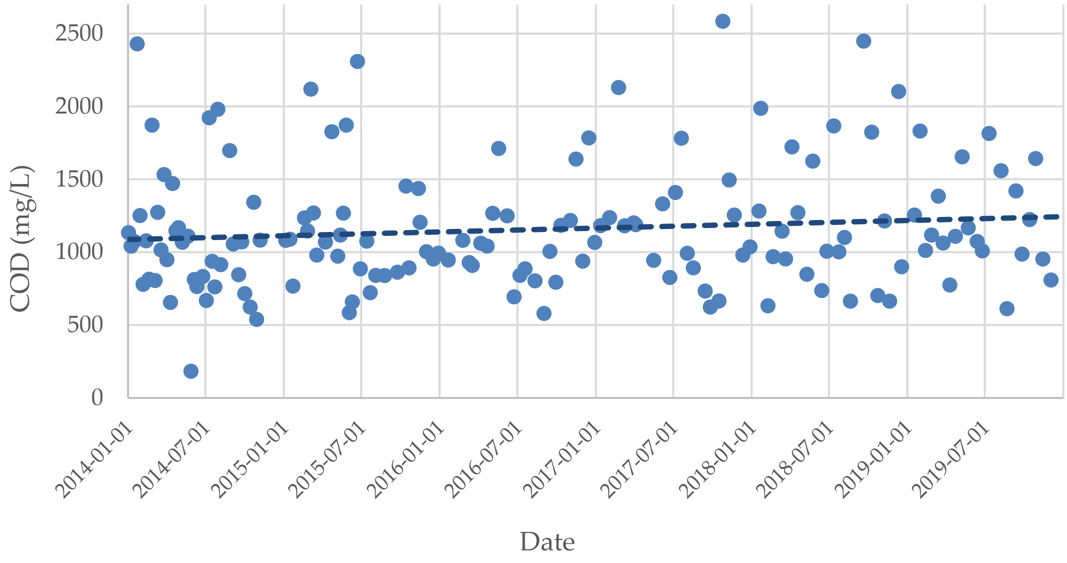

Figure S3.2. Evolution of COD in the influent of the NWWTS of Santa Lucía

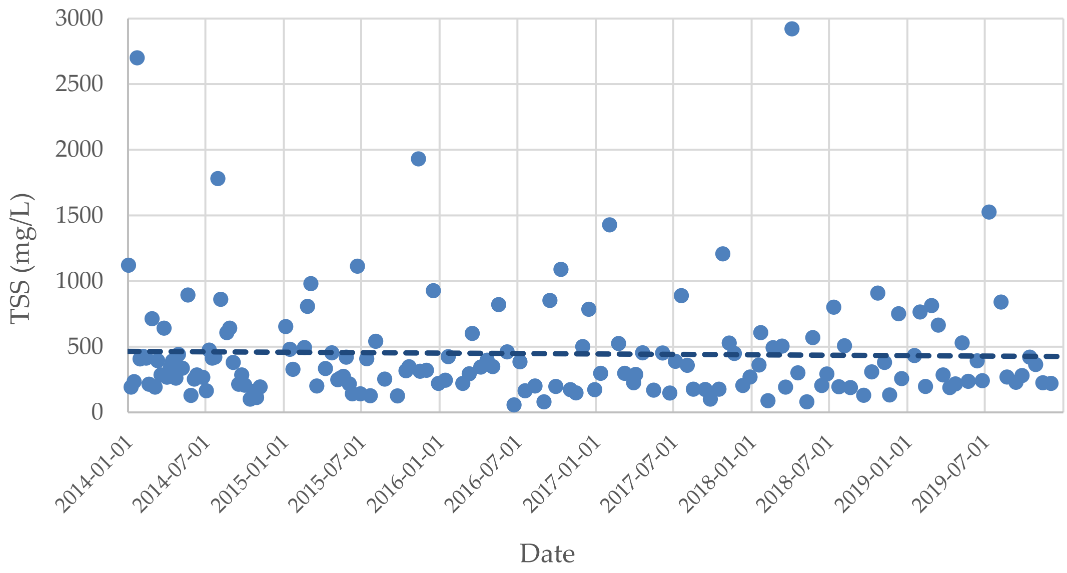

Figure S3.3. Evolution of TSS in the influent of the NWWTS of Santa Lucía

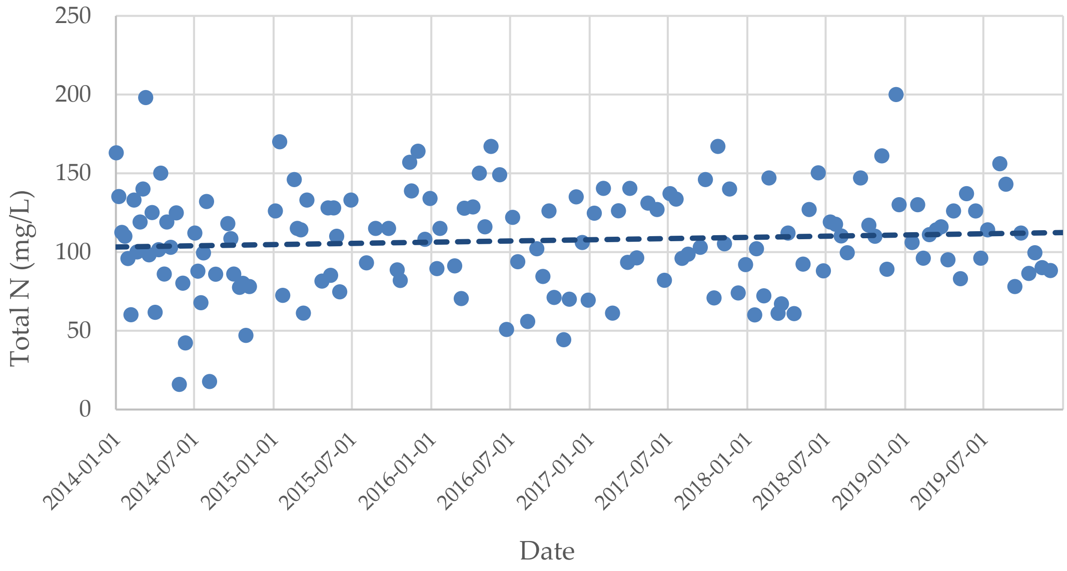

Figure S3.4. Evolution of total N in the influent of the NWWTS of Santa Lucía

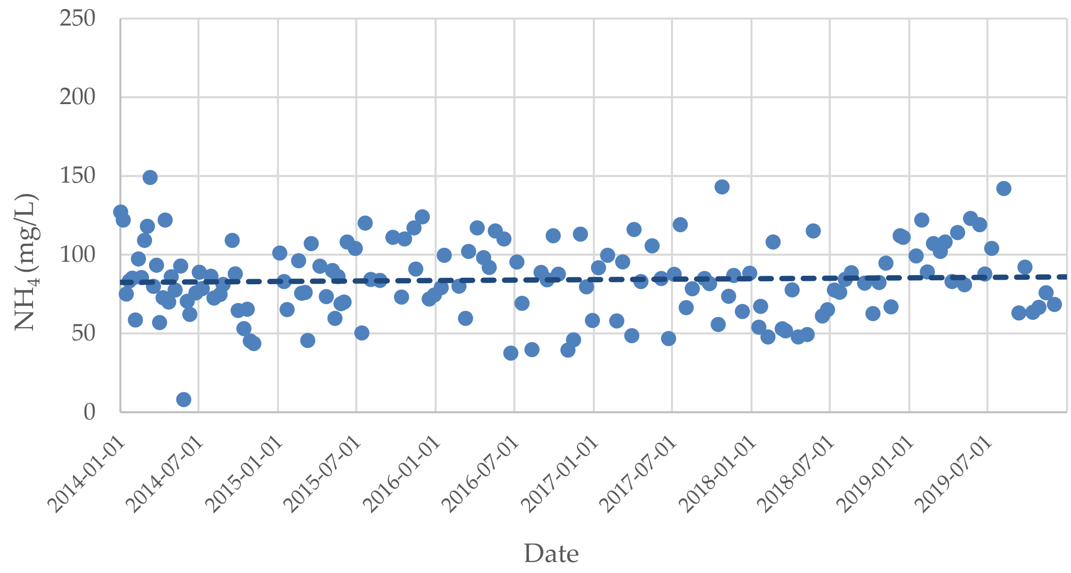

Figure S3.5. Evolution of NH_4 in the influent of the NWWTS of Santa Lucía

Primary treatment effluent

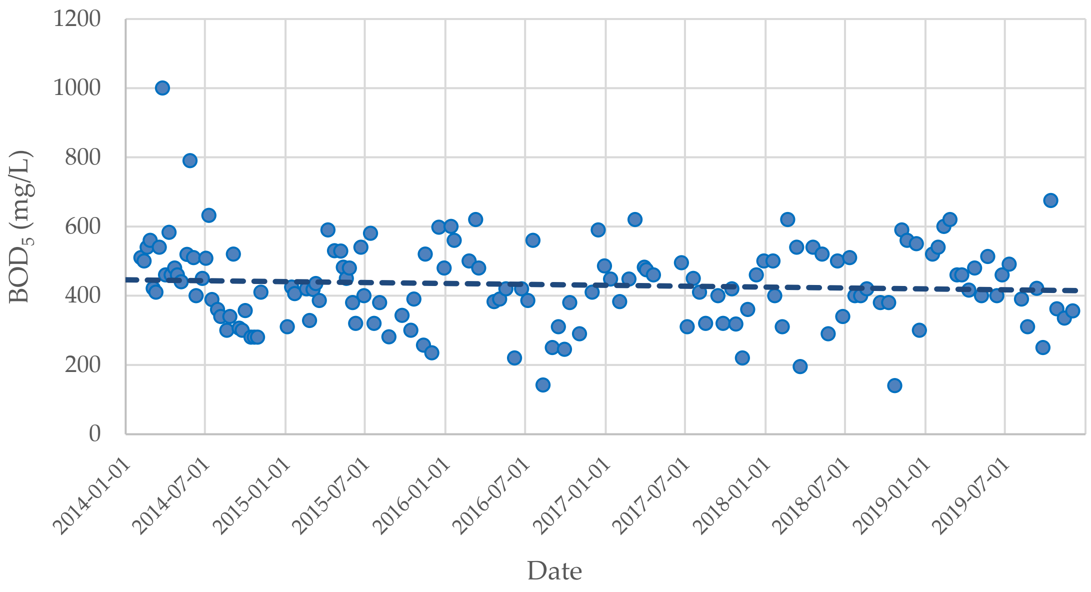

Figure S3.6. Evolution of BOD_5 in the primary treatment effluent

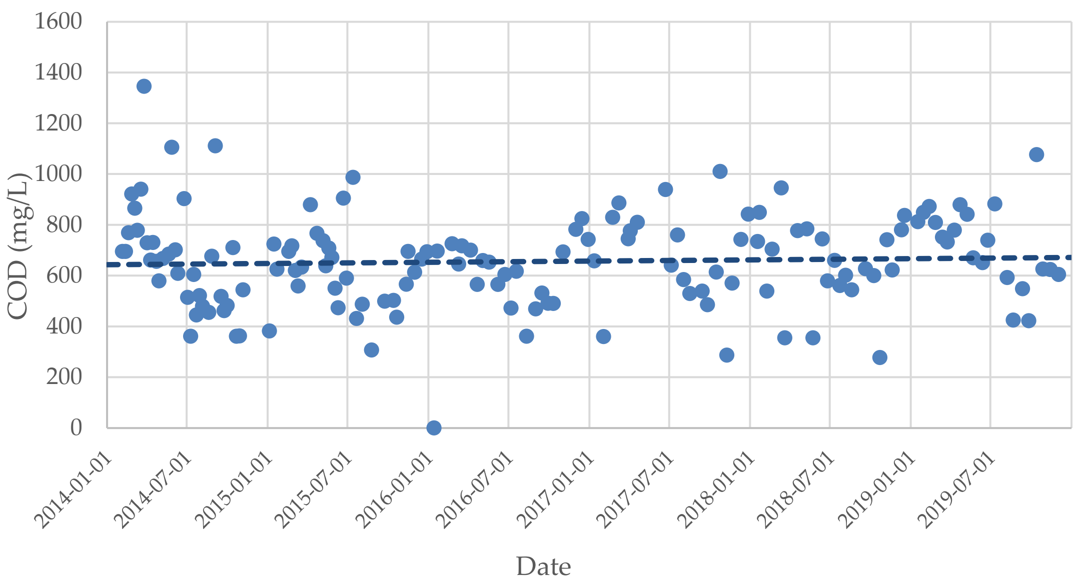

Figure S3.7. Evolution of COD in the primary treatment effluent

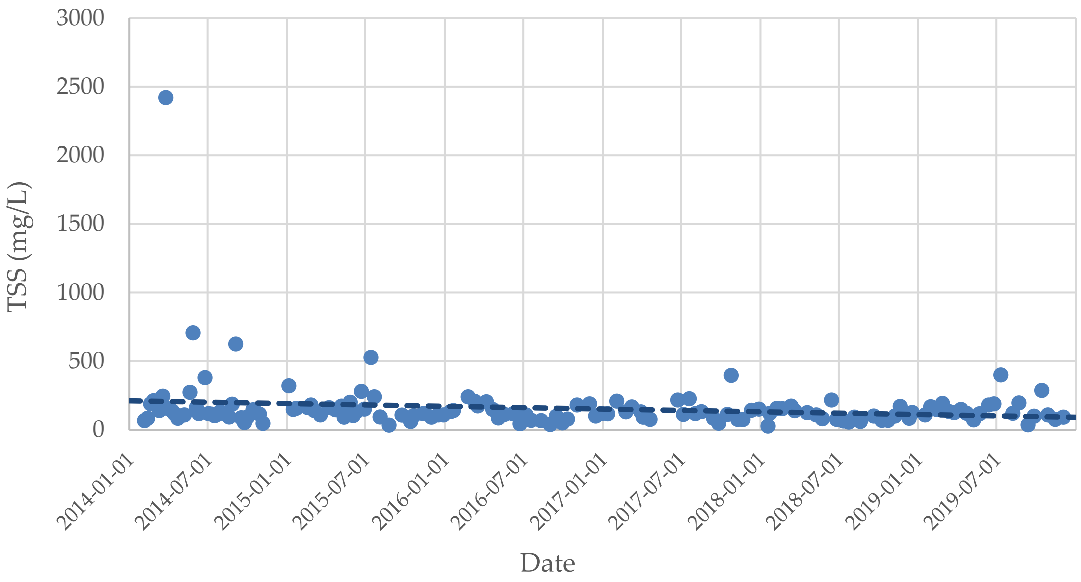

Figure S3.8. Evolution of TSS in the primary treatment effluent

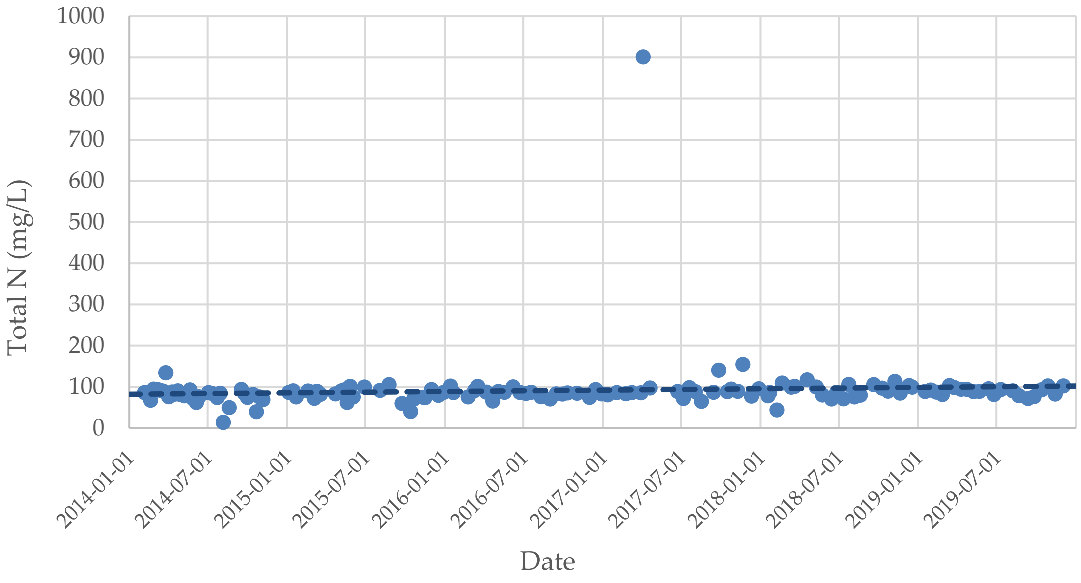

Figure S3.9. Evolution of total N in the primary treatment effluent

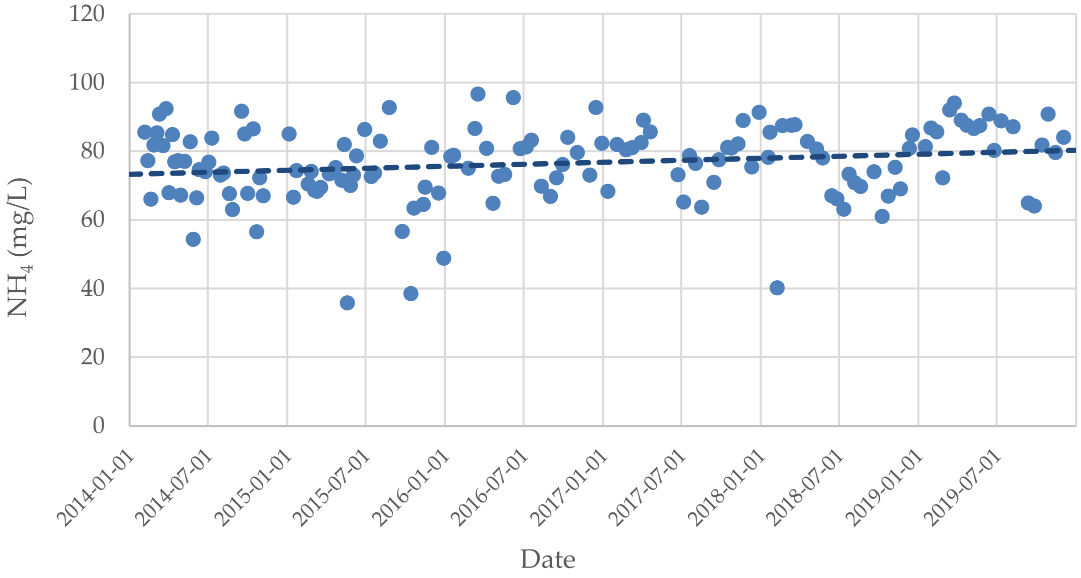

Figure S3.10. Evolution of NH_4 in the primary treatment effluent

<u>Secondary treatment effluent</u>

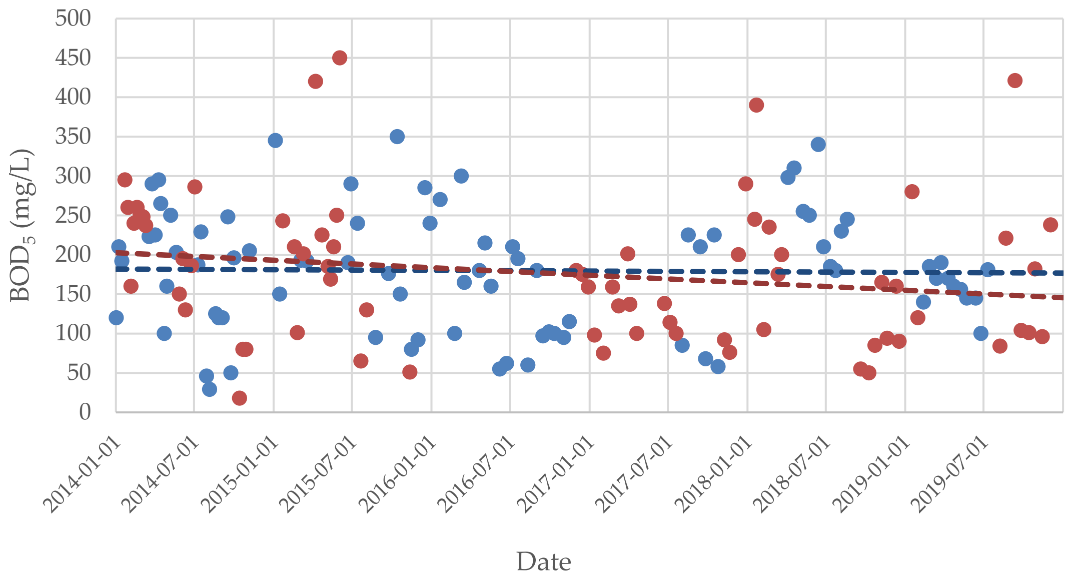

Figure S3.11. Evolution of BOD_5 in the effluent of the right (blue) and left (orange) VFCW

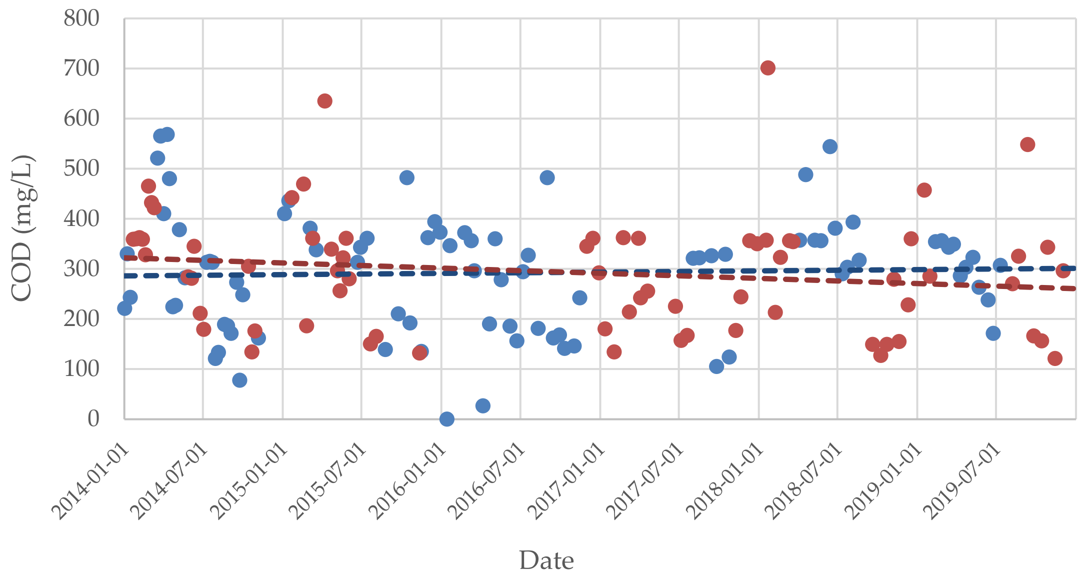

Figure S3.12. Evolution of COD in the effluent of the right (blue) and left (orange) VFCW

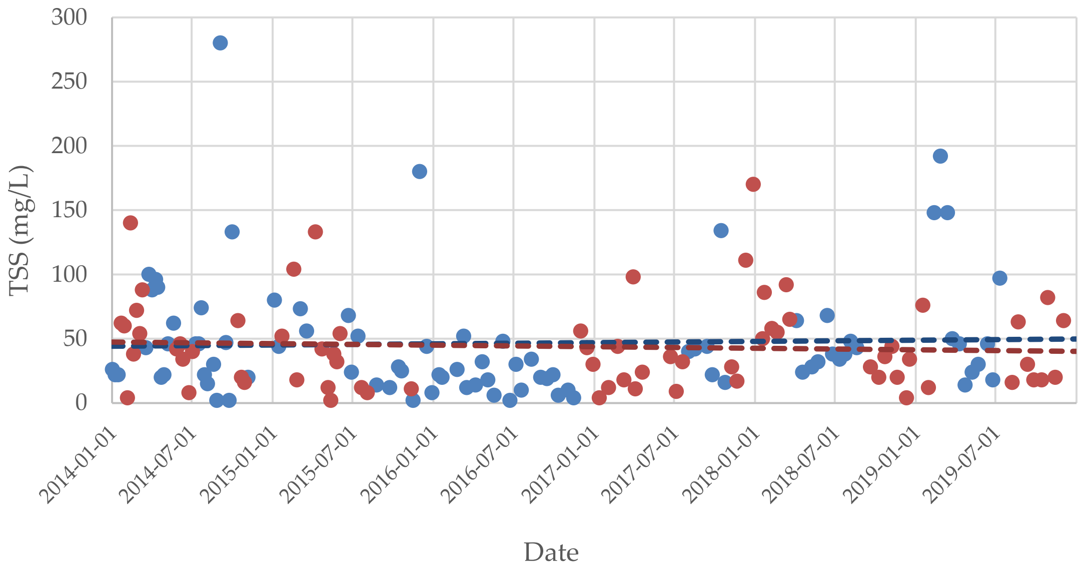

Figure S3.13. Evolution of TSS in the effluent of the right (blue) and left (orange) VFCW

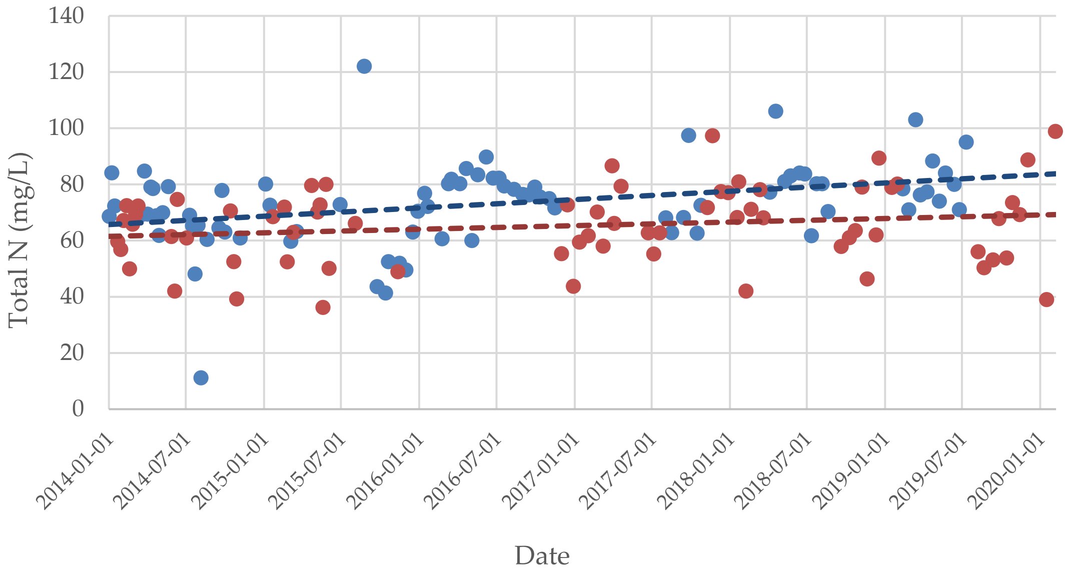

Figure S3.14. Evolution of total N in the effluent of the right (blue) and left (orange) VFCW

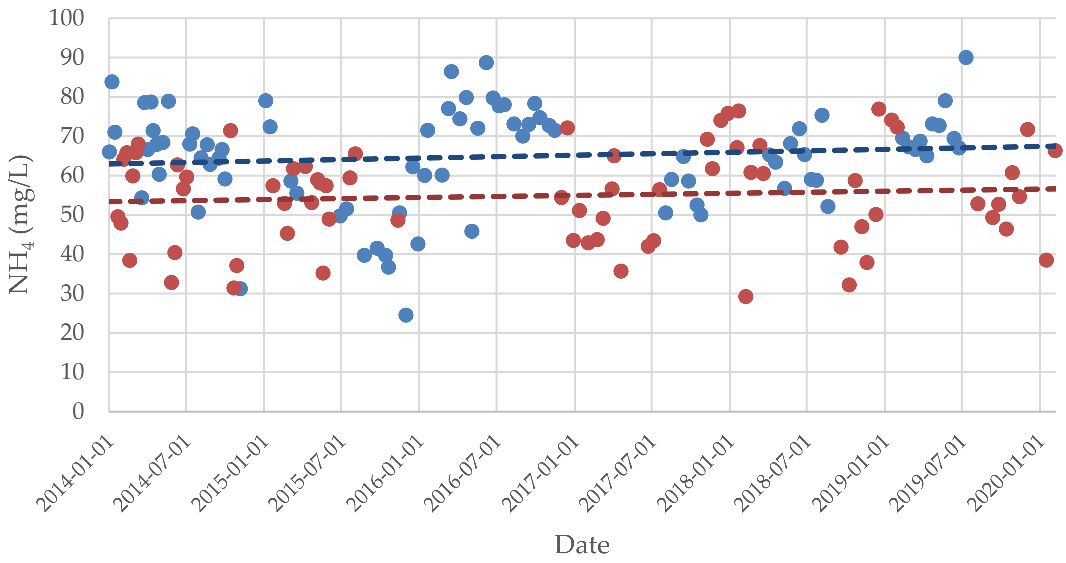

Figure S3.15. Evolution of NH_4 in the effluent of the right (blue) and left (orange) VFCW

Secondary treatment effluent

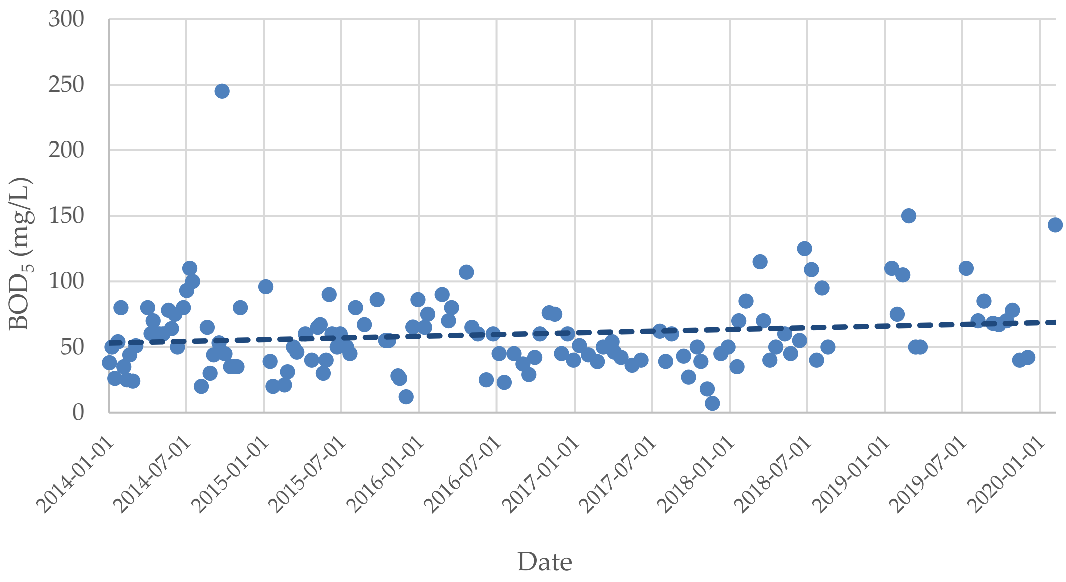

Figure S3.16. Evolution of BOD_5 in the effluent of the HFCW

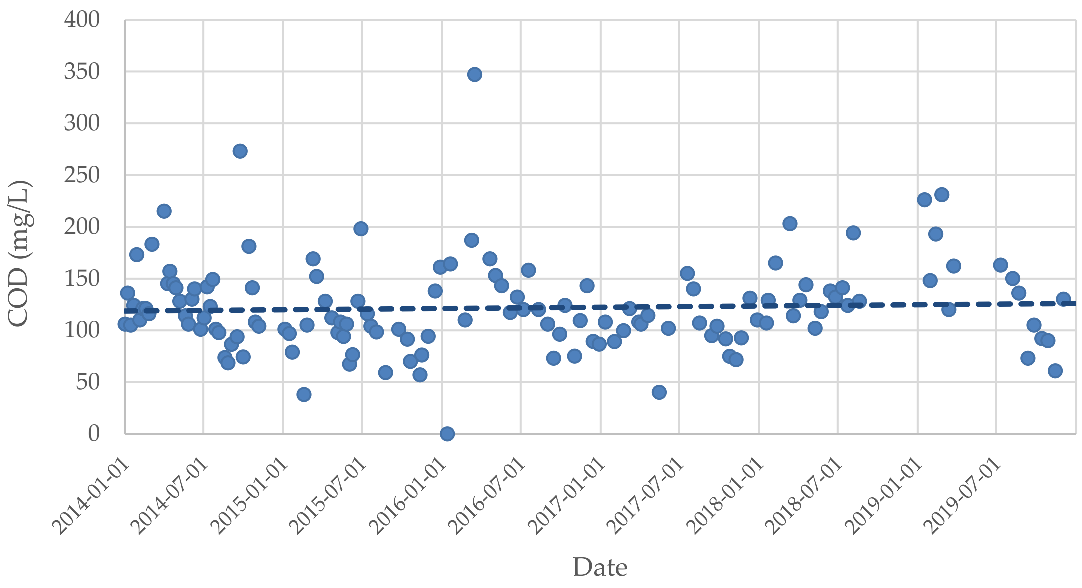

Figure S3.17. Evolution of COD in the effluent of the HFCW

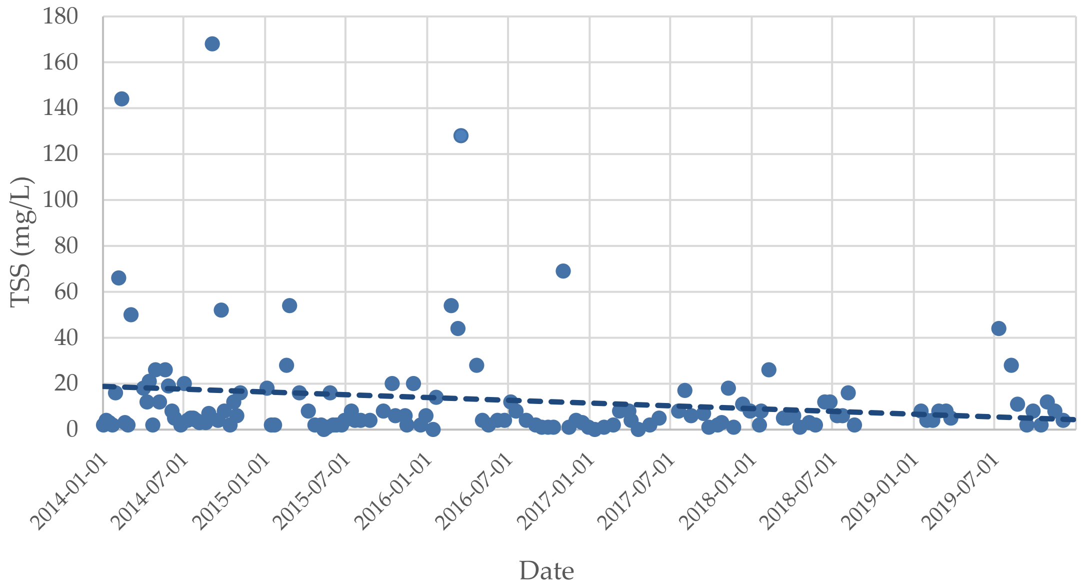

Figure S3.18. Evolution of TSS in the effluent of the HFCW

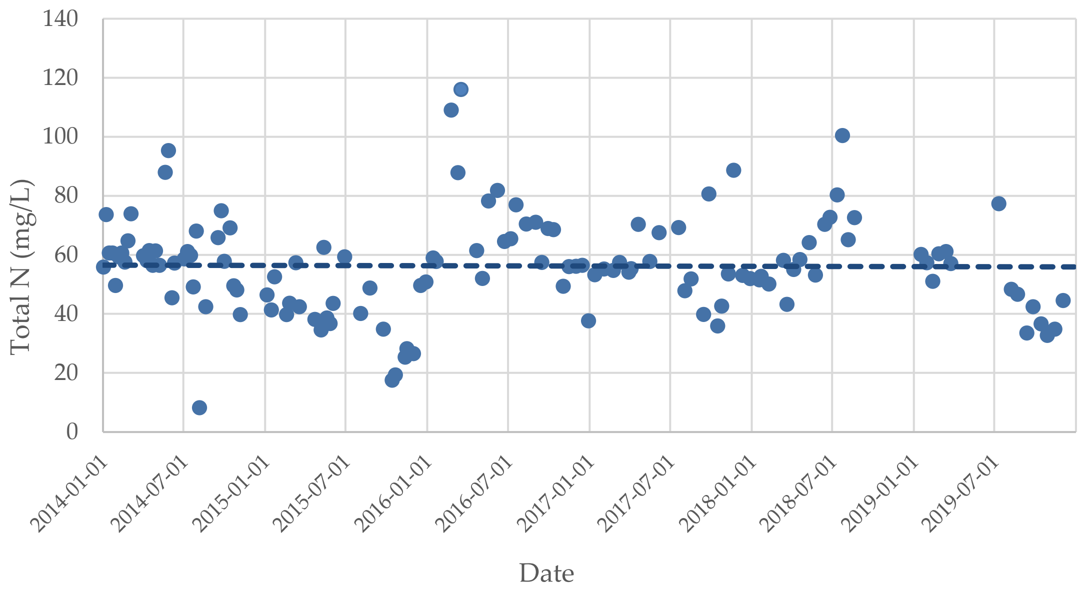

Figure S3.19. Evolution of total N in the effluent of the HFCW

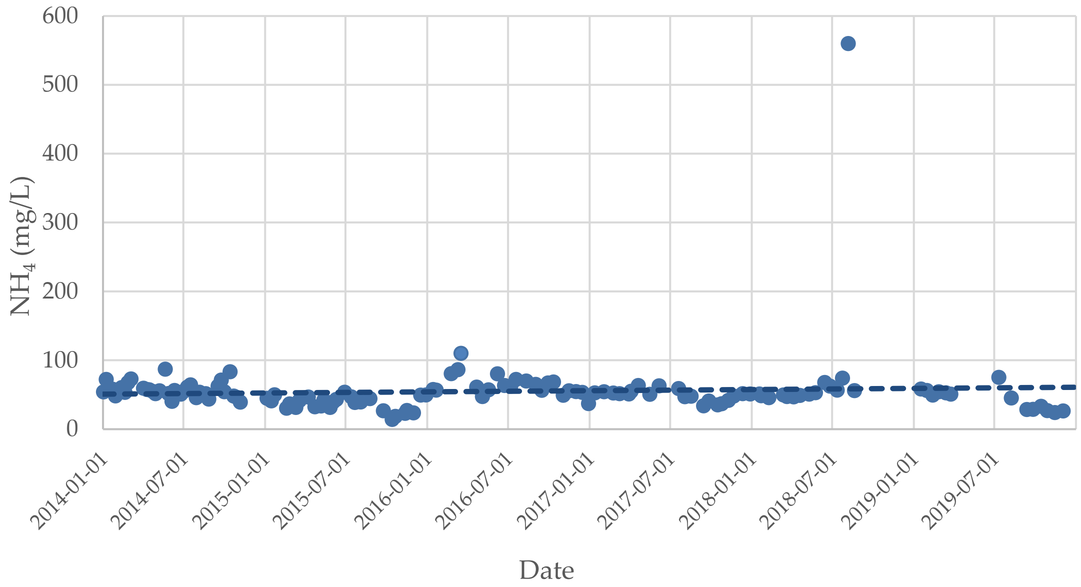

Figure S3.20. Evolution of NH_4 in the effluent of the HFCW
