# Supplementary material for: Long-Term Performance of a Hybrid-Flow Constructed Wetlands System for Urban Wastewater Treatment in Caldera de Tirajana (Santa Lucía, Gran Canaria, Spain)
Source: Int J Environ Res Public Health. 2022 Nov 11;19(22):14871. doi: 10.3390/ijerph192214871 (PMC9690933; doi:10.3390/ijerph192214871)
Supplement: Supplementary file 1 [file ijerph-19-14871-s001.zip › Figures S4.1 and S4.2.pdf]

Supplementary information S4

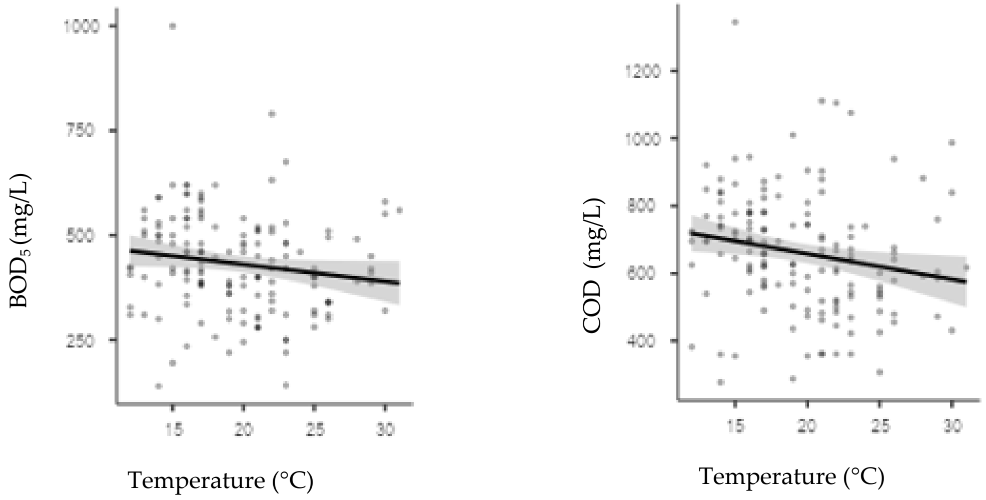

(**a**) (**b**)

Figure S4.1. Correlation between BOD_5 (**a**) and COD (**b**) in the primary treatment effluent and temperature

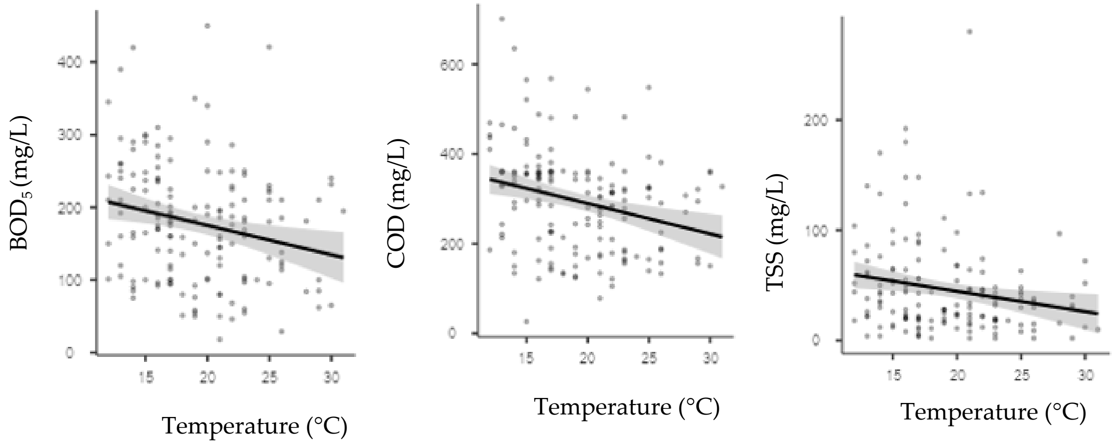

(**a**) (**b**) (**c**)

Figure S4.2. Relationship between BOD5 (**a**), COD (**b**) and TSS (**c**) and temperature in the effluent of the VFCWs
