# Supplementary material for: Long-Term Performance of a Hybrid-Flow Constructed Wetlands System for Urban Wastewater Treatment in Caldera de Tirajana (Santa Lucía, Gran Canaria, Spain)
Source: Int J Environ Res Public Health. 2022 Nov 11;19(22):14871. doi: 10.3390/ijerph192214871 (PMC9690933; doi:10.3390/ijerph192214871)
Supplement: Supplementary file 1 [file ijerph-19-14871-s001.zip › Table S5.1.pdf]

Supplementary information S5:

Table S5.1. Periods of activity of each VFCW (January 2014-December 2019)

RVFCW		Operating days: RVFCW	LVFCW		Operating days: LVFCW
01/01/2014	20/01/2014	19	20/01/2014	15/03/2014	54
15/03/2014	26/05/2014	72	26/05/2014	06/07/2014	41
06/07/2014	10/10/2014	96	10/10/2014	01/11/2014	22
01/11/2014	19/01/2015	79	19/01/2015	01/03/2015	41
01/03/2015	09/03/2015	8	09/03/2015	17/06/2015	100
17/03/2015	06/04/2015	20	06/04/2015	17/06/2015	72
17/06/2015	20/07/2015	33	20/07/2015	20/08/2015	31
20/08/2015	07/11/2015	79	07/11/2015	14/11/2015	7
14/11/2015	27/11/2016	379	27/11/2016	31/07/2017	246
31/07/2017	03/11/2017	95	03/11/2017	31/03/2018	148
31/03/2018	16/09/2018	169	16/09/2018	06/02/2019	143
06/02/2019	27/07/2019	171	27/07/2019	31/12/2019	157
	**TOTAL RVFCW**	**1 220 days**		**TOTAL LVFCW**	**1 062 days**
