# Supplementary material for: Long-Term Performance of a Hybrid-Flow Constructed Wetlands System for Urban Wastewater Treatment in Caldera de Tirajana (Santa Lucía, Gran Canaria, Spain)
Source: Int J Environ Res Public Health. 2022 Nov 11;19(22):14871. doi: 10.3390/ijerph192214871 (PMC9690933; doi:10.3390/ijerph192214871)
Supplement: Supplementary file 1 [file ijerph-19-14871-s001.zip › Tables S6.1 to S6.3.pdf]

Supplementary information S6

Table S6.1. Mean removal efficiencies of the different treatment stages of the Santa Lucía system, Gran Canaria (Spain) [24]

Treatment stages	Efficiency, %	BOD_5	COD	TSS	Total N	NH_4
Primary treatment	Septic tank	36	34	38	18	13
	Imhoff tank	-	-	-	-	-
**Overall primary treatment**		-	-	-	-	-
Secondary treatment	VFCW	71	64	76	33	34
	HFCW	49	41	63	21	15
**Overall secondary treatment**		88	81	97	54	40
**Overall system**		93	89	98	61	48

Equivalent inhabitants: 348.5 (Average inflow: 30 m^3/day; Influent average BOD_5 concentration: 697 mg/L).

Table S6.2. Mean removal efficiencies of the different treatment stages of the GEMMA group system, Barcelona (Spain) [33]

Treatment stages	Efficiency, %	BOD_5	COD	TSS	Total N	NH_4-N
**Overall primary treatment**		18.90	7.80	84.47	20.29	17.21
Secondary treatment	VFCW	80.45	67.72	48.00	57.93	73.76
	HFCW	38.46	22.95	84.62	44.74	54.72
**Overall secondary treatment**		87.97	75.13	92.00	76.75	88.12
**Overall system**		**90.24**	**77.07**	**98.76**	**81.47**	**90.16**

Note: Calculated on the basis of the input and output data of Table 2 of the paper.

Equivalent inhabitants: 0.5 (Average inflow: 0.2 m^3/day; Influent average BOD_5 concentration: 164 mg/L).

Table S6.3. Mean removal efficiencies of the different treatment stages of the CENTA system, Sevilla (Spain) [30]

Treatment stages	Efficiency, %	BOD_5	COD	TSS	Total N	NH_4-N
**Overall primary treatment**		48.09	45.45	65.85	3.48	-2.38
**Secondary treatment**	VFCW	95.10	84.35	91.84	66.60	75.87
	HFCW	50.00	34.78	0.00	53.41	47.12
**Overall secondary treatment**		97.55	89.80	91.84	84.44	87.24
**Overall system**		98.73	94.43	97.21	84.98	86.94

Note: Calculated on the basis of the input and output data (dry period) of Table 3 of the paper.

Equivalent inhabitants: 91.7 (Dry period: Average inflow: 14 m^3/day; Influent average BOD_5 concentration: 393 mg/L).
